# Adherence to the Canadian 24-hour movement guidelines and vision impairment in children and adolescents: a cross-sectional study

**DOI:** 10.3389/fmed.2025.1523640

**Published:** 2025-02-12

**Authors:** Haoxi Zhong, Huimin Zhu, Mingjie Jiang, Jingfeng Mu

**Affiliations:** Shenzhen Eye Hospital, Shenzhen Eye Medical Center, Southern Medical University, Shenzhen, China

**Keywords:** physical activity, screen time, sleep, public health, myopia, anisometropia, 24-hour movement, children and adolescents

## Abstract

**Objectives:**

To investigate the associations between adherence to the Canadian 24-hour movement guidelines—covering physical activity (PA), screen time (ST), and sleep duration (SD)—and vision impairment, specifically myopia and myopic anisometropia, among children and adolescents in Shenzhen, China.

**Methods:**

A cross-sectional study was conducted in 2022 with 4,649 participants. Adherence to the guidelines was assessed using self-reported PA, ST, and SD measures, while vision impairment was clinically evaluated. Logistic regression models were used to analyze the associations, adjusting for sociodemographic factors.

**Results:**

Among the participants, 48.63% were diagnosed with myopia and 11.01% had myopic anisometropia. Meeting the ST guideline was associated with a reduced risk of myopia (aOR = 0.86, 95% CI = 0.76-0.98) and myopic anisometropia (aOR = 0.78, 95% CI = 0.64-0.95). Meeting both PA and ST guidelines further reduced the odds of myopia (aOR = 0.73, 95% CI = 0.56-0.97) and myopic anisometropia (aOR = 0.60, 95% CI = 0.41-0.89). Meeting all three guidelines (PA, ST, and SD) significantly reduced the odds of myopia (aOR = 0.71, 95% CI: 0.53–0.93) and showed a trend toward reduced risk of anisometropia (aOR = 0.69, 95% CI: 0.47–1.02), compared to those who met none. Meeting two guidelines also significantly reduced the risk of myopia (aOR = 0.76, 95% CI: 0.59–0.97) and anisometropia (aOR = 0.71, 95% CI: 0.51–1.00).

**Conclusion:**

Adherence to the 24-hour movement guidelines, particularly meeting the ST and PA recommendations, was associated with a lower risk of myopia and myopic anisometropia. These findings highlight the importance of promoting balanced lifestyle behaviors, such as limiting screen time and encouraging physical activity, to mitigate vision impairment among children and adolescents.

## Introduction

Vision impairment, including myopia and anisometropia, is a rapidly growing public health concern, particularly among children and adolescents worldwide (1). Myopia, commonly known as nearsightedness, is characterized by the elongation of the eyeball, which causes distant objects to appear blurry (2). If left untreated, can lead to high myopia, which significantly increases the risk of sight-threatening complications such as glaucoma, retinal detachment, and macular degeneration (2–6). The prevalence of myopia has surged in recent decades, especially in East Asian countries, where urbanization and changes in lifestyle have contributed to an “epidemic” of vision impairment in younger populations (7–9).

Anisometropia, another common type of vision impairment, is defined as an interocular difference in refractive power of ≥1.00 diopter (10). This condition can impair binocular vision and depth perception, particularly in children, and may lead to amblyopia (“lazy eye”) if left untreated (11–13). Among the subtypes of anisometropia, myopic anisometropia—characterized by both eyes being myopic or one eye being myopic and the other emmetropic—is the most prevalent form, with prevalence rates ranging from 45 to 56% according to previous studies (13–16). Understanding the factors that contribute to anisometropia, especially its myopic subtype, is essential for developing targeted prevention and intervention strategies. Unlike general myopia, myopic anisometropia can lead to additional clinical complications, such as reduced binocular vision, impaired stereopsis, and an increased risk of amblyopia (10–12). These functional deficits underscore the importance of identifying modifiable lifestyle factors to inform precise and effective interventions.

A growing body of research has identified several environmental and behavioral factors associated with the development and progression of myopia (17–19). Among these, lifestyle behaviors such as physical activity (17, 20–22), screen time (17, 23, 24), and sleep duration (25, 26) have been increasingly recognized as potential modifiable risk factors. These behaviors are closely interconnected, and failing to meet all of the guidelines—specifically by exceeding screen time limits, engaging in insufficient physical activity, and not getting adequate sleep—poses the greatest risk to children’s vision, particularly in urban environments where academic pressures and technological advancements shape their daily routines (17, 24).

To address these lifestyle challenges, the Canadian 24-Hour Movement Guidelines for Children and Youth provide an evidence-based framework that integrates recommendations for physical activity, screen time, and sleep to promote a healthy balance across a 24-h period (27). Specifically, the guidelines recommend at least 60 min of moderate-to-vigorous physical activity per day, no more than 2 h of recreational screen time, and sufficient sleep based on age (27, 28). These guidelines are widely promoted for their benefits in improving physical and mental health (29–36), but limited research has explored their potential role in protecting against vision impairment (37), particularly myopia and anisometropia.

While some studies suggest that increased physical activity, especially outdoor activity, may have a protective effect against myopia (20, 21, 38–40), others highlight the detrimental effects of excessive screen time on eye health (18, 23, 24). The relationship between sleep duration and myopia remains more controversial, with some studies reporting a link between poor sleep quality and increased myopia risk, while others find no significant association (25). These diverging hypotheses underscore the need for a comprehensive investigation into the combined effects of physical activity, screen time, and sleep duration on children’s vision.

This cross-sectional study aims to address this gap by examining the associations between adherence to the Canadian 24-Hour Movement Guidelines and vision impairment, specifically myopia and anisometropia, in children and adolescents in Shenzhen, China. By investigating the independent and combined effects of meeting the guidelines, this study provides critical insights into how modern lifestyle behaviors may influence visual health. The findings have the potential to inform public health strategies and interventions designed to reduce the growing burden of vision impairment in young populations.

## Materials and methods

### Study participants

This cross-sectional study was conducted among schoolchildren aged 6–13 years in Shenzhen, China, between March 3 and May 3, 2022. A total of 5,140 parents consented to participate in the study, completing an online survey about their children, who also underwent comprehensive eye examinations.

The inclusion criteria were: (1) children aged 6–13 years enrolled in primary and middle schools in Shenzhen, China; (2) participants who provided informed consent from their parents or legal guardians; (3) completion of both the online questionnaire and school-based eye examinations. The exclusion criteria were: (1) participants with incomplete or missing data on key variables such as physical activity, screen time, sleep duration, or vision impairment status; (2) children with known eye diseases or conditions unrelated to refractive errors (e.g., congenital cataracts, glaucoma) that could affect vision outcomes; (3) children with subtypes of anisometropia other than myopic anisometropia were also excluded; (4) participants who were unable to cooperate with the eye examinations.

After applying the inclusion and exclusion criteria, a total of 4,649 participants were included in the final analysis. The study protocol was reviewed and approved by the Ethics Committee of Shenzhen Eye Hospital (Approval No.: 20201230–06). Written informed consent was obtained from all legal guardians of the participants prior to enrollment.

### Outcomes

The primary outcomes of interest were myopia and myopic anisometropia, both assessed through school-based vision screening by trained optometrists following the Chinese Health Standard (WS/T 663–2020). The vision screening involved two main assessments: visual acuity and refractive error.

Visual acuity was measured using a standard visual acuity chart (Eye Vision 1,603–01, China). It was recorded in decimal notation and subsequently transformed into a 5-grade notation for statistical analysis, as outlined in the Chinese Health Standard (WS/T 663–2020). This system, developed in China, is widely used for vision screening in school settings and can be equivalently mapped to other notation systems, including Snellen decimal and logMAR (Table 1).

**Table 1:** Transformation among 5-grade notation, decimal notation and LogMAR notation.

Notation systems	Equivalence													
5-Grade	3.0	4.0	4.1	4.2	4.3	4.4	4.5	4.6	4.7	4.8	4.9	5.0	5.1	5.2
LogMAR	2.0	1.0	0.9	0.8	0.7	0.6	0.5	0.4	0.3	0.2	0.1	0.0	−0.1	−0.2
Decimal	0.01	0.10	0.12	0.16	0.20	0.25	0.32	0.40	0.50	0.63	0.80	1.00	1.25	1.63

Non-cycloplegic refraction was conducted using an autorefractor (NIDEK AR-1, Japan). Myopia was defined as a spherical equivalent refraction (SER) of less than −0.50 diopters in either eye, along with an uncorrected visual acuity of less than 5.0, or as wearing orthokeratology lenses (a form of myopia control therapy) (41). The classification of anisometropia in this study was based on the framework proposed by Nunes et al. (42). Myopic anisometropia was defined as an interocular difference in spherical equivalent refraction (SER) of ≥1.00D, with both eyes being myopic or one eye being myopic and the other emmetropic. By analyzing myopic anisometropia as a distinct subgroup, this study seeks to uncover specific lifestyle patterns and risk factors that may differ from general myopia, thereby providing deeper insights into the etiology and prevention of interocular refractive disparities. Participants with meridional anisometropia were excluded from the analysis. The prevalence of different subtypes of anisometropia among the study participants is presented in the Supplementary material, along with sensitivity analysis results examining the associations of physical activity (PA), screen time (ST), and sleep duration (SD) with these subtypes.

### Questionnaire

An eye health questionnaire was developed to collect data on key exposures and potential covariates. This included demographic information (age, sex), lifestyle factors (physical activity, screen time, sleep duration), and clinical history (parental myopia, prematurity, breastfeeding). Additional information was collected on body mass index (BMI), posture during reading and writing, living area per person, and per capita household income. Parents or legal guardians completed the questionnaire on behalf of the children.

The reliability of the questionnaire was assessed by administering it twice, with a one-day interval, to the parents or guardians of 100 randomly selected students. The results demonstrated high reliability, with correlation coefficients exceeding 0.95 for all items, indicating excellent consistency.

### Adherence to 24-hour movement guidelines

Adherence to the 24-hour movement guidelines was assessed using a detailed questionnaire completed by the parents or guardians of the participants. The guidelines included three components: physical activity (PA), screen time (ST), and sleep duration (SD).

Physical Activity (PA): adherence was defined as engaging in at least 60 min of moderate-to-vigorous physical activity per day. Parents reported their child’s daily physical activity levels, including participation in school-based activities, organized sports, and unstructured outdoor play.

Screen Time (ST): adherence was defined as limiting recreational screen time (e.g., using tablets, smartphones, computers, and watching TV) to 2 hours or less per day. The questionnaire collected information on the average time spent using electronic devices for non-educational purposes over the past week.

Sleep Duration (SD): adequate sleep was defined based on age-specific recommendations: 9–11 h per night for children aged 6–13 years. Parents reported their child’s average sleep duration, including naps and nighttime sleep, over the past week.

### Covariates

Several covariates were included in the analysis to account for potential confounding factors that may influence the relationship of adherence to the 24-hour movement guidelines with the risk of myopia and anisometropia. Age was treated as a continuous variable, while sex was categorized as male or female. Parental myopia was self-reported and classified as present if either parent had myopia. Parental education level was grouped into four categories: junior middle school or below, high school or equivalent, bachelor or equivalent and master or above. Family socioeconomic status was assessed using per capita monthly household income, categorized into five distinct ranges. These variables were selected based on their known associations with vision impairment and lifestyle behaviors, and they were incorporated into the statistical models to adjust for potential confounding, ensuring robust estimates of the associations under investigation.

### Statistical analyses

All statistical analyses were conducted using R (version 4.4.1). Descriptive statistics were used to summarize the sociodemographic characteristics of the participants and their adherence to the Canadian 24-hour movement guidelines. Categorical variables were expressed as frequencies and percentages, while continuous variables, such as age, were presented as means and standard deviations (SD). The primary outcomes of interest were myopia and anisometropia, both measured as binary variables (yes/no). The primary exposure variables were adherence to the PA, ST, and SD components of the 24-hour movement guidelines, which were also treated as binary variables (yes/no), number of guidelines met (0/1/2/3). Additional analyses included combined adherence to one, two, or all three guidelines.

To investigate the associations between adherence to the movement guidelines and the odds of vision impairment (myopia and anisometropia), logistic regression analysis was fitted for each outcome. Crude odds ratios (OR) were first calculated to assess unadjusted associations. Multivariable logistic regression models were then used to calculate adjusted odds ratios (aOR) and 95% confidence intervals (CI), controlling for potential confounders, including age, gender, parental myopia, parental education, and family income. Separate models were built for each guideline (PA, ST, SD) as well as for combinations of the guidelines to assess their independent and combined effects on vision impairment.

Goodness-of-fit for the logistic regression models was evaluated using the *Hosmer-Lemeshow* test, and statistical significance was set at *p* < 0.05. All tests were two-sided. In addition, sensitivity analyses were conducted to evaluate the effects of PA, ST, and SD on the different subtypes of anisometropia, with detailed results presented in the Supplementary materials.

## Results

### Sample characteristics

A total of 4,649 children and adolescents aged 6–13 years were included in the final analysis. The sociodemographic characteristics of the sample are presented in Table 2. The mean age of the participants was 9.6 years (SD = 1.8). The prevalence of myopia and anisometropia in the sample was 48.63 and 11.01%, respectively. Myopia prevalence was significantly associated with age (*p* < 0.001), with higher rates in older age groups. Gender was also significantly associated, with girls showing a higher prevalence of myopia (*p* < 0.001) and anisometropia (*p* = 0.019) compared to boys.

**Table 2:** Descriptive statistics of sociodemographic variables.

Sociodemographic variables	Myopia		Statistical values	*p*-value	Myopic anisometropia		Statistical values	*p*-value
	Yes	No			Yes	No		
Age	2,261 (48.63)	2,388 (51.37)	24.5555	<0.001	512 (11.01)	4,137 (88.99)	9.8725	<0.001
(mean = 9.6, SD = 1.8)								
Gender								
Boys	1,178 (25.34)	1,384 (29.77)	15.762	<0.001	257 (5.53)	2,305 (49.58)	6.394	0.019
Girls	1,083 (23.30)	1,004 (21.60)			255 (5.49)	1832 (39.41)		
Grades								
Grade 1	269 (5.79)	827 (17.79)	668.183	<0.001	69 (1.48)	1,027 (22.09)	112.219	<0.001
Grade 2	276 (5.94)	534 (11.49)			56 (1.20)	754 (16.22)		
Grade 3	437 (9.40)	430 (9.25)			83 (1.79)	784 (16.86)		
Grade 4	336 (7.23)	236 (5.08)			65 (1.40)	507 (10.91)		
Grade 5	495 (10.65)	225 (4.84)			115 (2.47)	605 (13.01)		
Grade 6	448 (9.64)	136 (2.93)			124 (2.67)	460 (9.89)		
Paternal myopia								
Yes	786 (16.91)	682 (14.67)	23.622	<0.001	163 (3.51)	1,305 (28.07)	0.96	0.920
No	1,475 (31.73)	1706 (36.70)			349 (7.51)	2,832 (60.92)		
Maternal myopia								
Yes	946 (20.35)	752 (16.18)	58.65	<0.001	188 (4.04)	1,510 (32.48)	0.015	0.923
No	1,315 (28.29)	1,636 (35.19)			324 (6.97)	2,627 (56.51)		
Parental education								
Junior middle school or below	466 (10.02)	457 (9.83)	10.324	0.016	114 (2.45)	809 (17.40)	2.202	0.510
High school or equivalent	573 (12.33)	543 (11.68)			123 (2.65)	993 (21.36)		
Bachelor or equivalent	1,125 (24.20)	1,281 (27.55)			253 (5.44)	2,153 (46.31)		
Master or above	97 (2.09)	107 (2.30)			22 (0.47)	182 (3.91)		
Family monthly income per capita								
<2000	76 (1.63)	79 (1.70)	10.158	0.038	17 (0.37)	138 (2.97)	4.204	0.379
2000–5,000	461 (9.92)	455 (9.79)			111 (2.39)	805 (17.32)		
5,000–10,000	787 (16.93)	764 (16.43)			188 (4.04)	1,363 (29.32)		
10,000–20,000	573 (12.33)	627 (13.49)			122 (2.62)	1,078 (23.19)		
>20,000	364 (7.83)	463 (9.96)			74 (1.59)	753 (16.20)		
Living spaces per person(m^2^)								
<10	258 (5.55)	276 (5.94)	1.583	0.663	66 (1.42)	468 (10.07)	0.989	0.619
10–20	648 (13.94)	663 (14.26)			148 (3.18)	1,163 (25.02)		
21–30	584 (12.56)	594 (12.78)			129 (2.77)	1,049 (22.56)		
>30	771 (16.58)	855 (18.39)			169 (3.64)	1,457 (31.34)		

SD = standard deviation.

Significant associations were observed between myopia prevalence and school grade (*p* < 0.001), with higher grades showing increased rates. Paternal and maternal myopia were significantly associated with myopia in children (*p* < 0.001 for both), but no significant association was found for anisometropia (*p* = 0.920 for paternal myopia; *p* = 0.923 for maternal myopia). Parental education level also showed a significant relationship with myopia (*p* = 0.016) but not with anisometropia. Family income per capita per month was significantly associated with myopia (*p* = 0.038), while living space per person did not show significant associations with either outcome.

### Adherence to 24-hour movement guidelines

**Figure 1:**
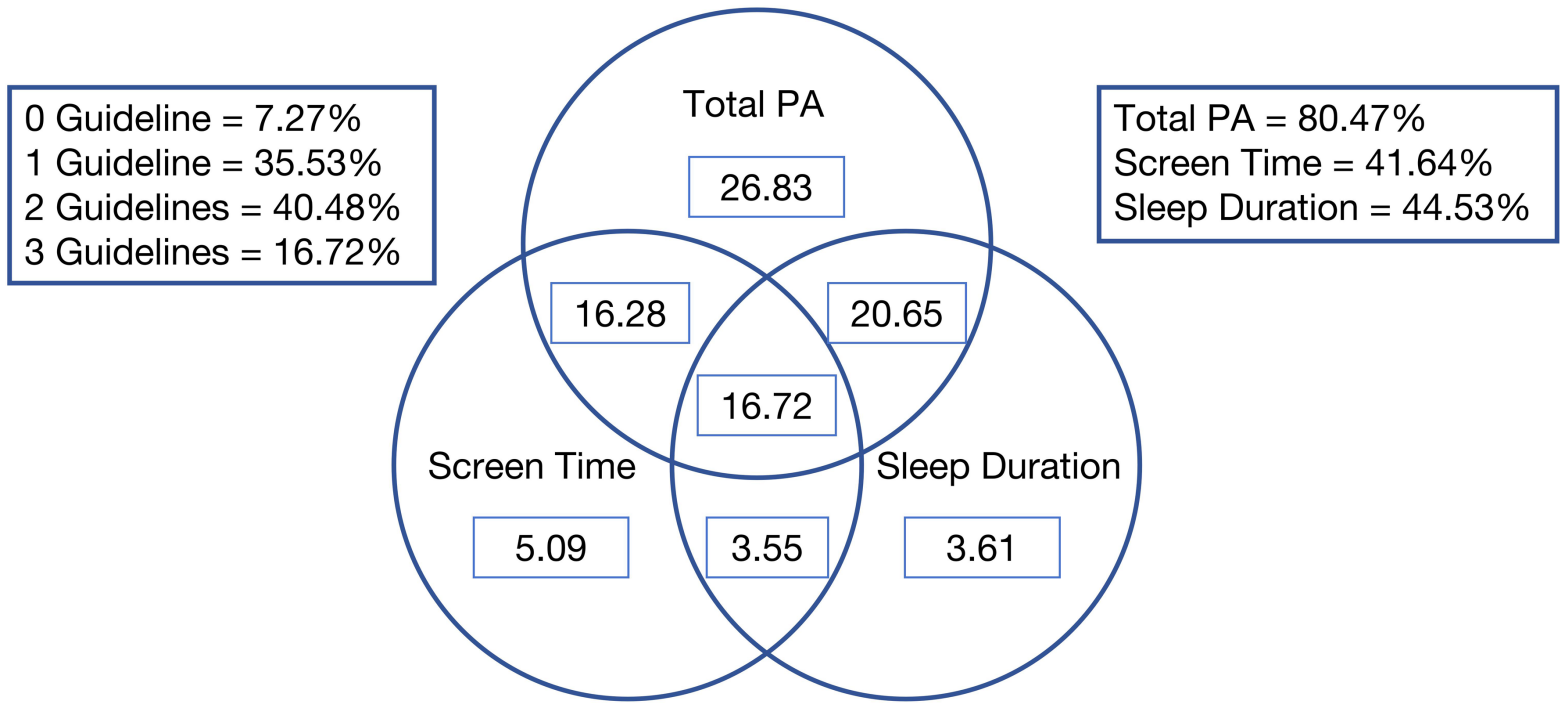
Venn diagram showing the proportion (%) of participants meeting the 24-hour movement guidelines.

The adherence rates to the 24-hour movement guidelines are shown in Table 3. A total of 80.47% of participants met the physical activity (PA) guideline, 41.64% met the screen time (ST) guideline, and 44.53% met the sleep duration (SD) guideline. Regarding adherence to the guidelines in combination, 7.27% of participants did not meet any of the guidelines, while 35.53% met one guideline, 40.48% met two, and 16.72% met all three guidelines. Specifically, 26.83% met only the PA guideline, 5.09% met only the ST guideline, and 3.61% met only the SD guideline. Figure 1 illustrates the proportion of participants meeting one, two, or all three guidelines.

**Table 3:** Descriptive statistics of adherence to 24-hour movement guidelines and vision impairment.

Exposures	Myopia		*χ* ^2^	*p*-value	Myopic anisometropia		*χ* ^2^	*p*-value
	Yes	No			Yes	No		
PA								
Yes	1780 (38.29)	1961 (42.18)	9.324	0.004	397 (8.54)	3,344 (71.93)	2.076	0.15
No	481 (10.35)	427 (9.18)			115 (2.47)	793 (17.06)		
ST								
Yes	843 (18.13)	1,093 (23.51)	35.869	<0.001	173 (3.72)	1763 (37.92)	11.533	0.001
No	1,418 (30.50)	1,295 (27.86)			339 (7.29)	2,374 (51.06)		
SD								
Yes	944 (20.31)	1,126 (24.22)	13.647	<0.001	220 (4.73)	1850 (39.79)	0.001	0.98
No	1,317 (28.33)	1,262 (27.15)			292 (6.28)	2,287 (49.19)		
Guidelines met								
None	201 (4.32)	137 (2.95)	56.141	<0.001	54 (1.16)	284 (6.11)	16.014	0.025
PA only	660 (14.20)	587 (12.63)			148 (3.18)	1,099 (23.64)		
ST only	115 (2.47)	122 (2.62)			25 (0.54)	212 (4.56)		
SD only	94 (2.02)	74 (1.59)			23 (0.49)	145 (3.12)		
PA and ST	341 (7.33)	416 (8.95)			65 (1.40)	692 (14.88)		
PA and SD	463 (9.96)	497 (10.69)			114 (2.45)	846 (18.20)		
ST and SD	71 (1.53)	94 (2.02)			13 (0.28)	152 (3.27)		
All	316 (6.80)	461 (9.92)			70 (1.51)	707 (15.21)		
Number of guidelines met								
None	201 (4.32)	137 (2.95)	51.327	<0.001	54 (1.16)	284 (6.11)	8.626	0.035
One	869 (18.69)	783 (16.84)			196 (4.22)	1,456 (31.32)		
Two	875 (18.82)	1,007 (21.66)			192 (4.13)	1,690 (36.35)		
Three	316 (6.80)	461 (9.92)			70 (1.51)	707 (15.21)		

PA = physical activity; ST = screen time; SD = sleep duration.

### Association between meeting 24-hour movement guidelines and vision impairment

Tables 4, 5 present the relationship between adherence to the 24-hour movement guidelines and vision impairment, adjusted for age, gender, parental myopia, parental education, and family income. Table 4 shows the independent effects of meeting each guideline on the odds of developing myopia and anisometropia. Participants who met the screen time guideline had a significantly reduced risk of myopia (adjusted odds ratio [aOR] = 0.86, 95% confidence interval [CI] = 0.76–0.98, *p* = 0.021) and anisometropia (aOR = 0.78, 95% CI = 0.64–0.95, *p* = 0.015). Adherence to the physical activity guideline was not significantly associated with myopia (aOR = 0.87, 95% CI = 0.74–1.01, *p* = 0.069) or anisometropia (aOR = 0.88, 95% CI = 0.70–1.10, *p* = 0.3). Sleep duration was not significantly associated with either myopia (aOR = 0.94, 95% CI = 0.83–1.06, *p* = 0.3) or anisometropia (aOR = 1.04, 95% CI = 0.86–1.25, *p* = 0.7).

**Table 4:** Association between meeting 24-hour movement guidelines and vision impairment^a^.

	Myopia			Myopic Anisometropia		
	aOR	95%CI	*p*-value	aOR	95%CI	*p*-value
PA	0.87	0.74, 1.01	0.069	0.88	0.70, 1.10	0.300
ST	0.86	0.76, 0.98	0.021	0.78	0.64, 0.95	0.015
SD	0.94	0.83, 1.06	0.300	1.04	0.86, 1.25	0.700

^aAdjusted for age, gender, parental myopia, parental education and family income.^

PA = physical activity; ST = screen time; SD = sleep duration; aOR = adjusted odds ratio; CI = confidence interval.

**Table 5:** Combined effects of meeting 24-hour movement guidelines on vision impairment^a^.

	Myopia			Myopic anisometropia		
	aOR	95%CI	*p*-value	aOR	95%CI	*p*-value
Guidelines met						
None	Reference			Reference		
PA only	0.83	0.64, 1.08	0.200	0.76	0.54, 1.07	0.110
ST only	0.79	0.55, 1.13	0.200	0.71	0.42, 1.17	0.200
SD only	1.05	0.70, 1.56	0.800	0.93	0.54, 1.57	0.800
ST and PA	0.73	0.56, 0.97	0.029	0.60	0.41, 0.89	0.011
SD and PA	0.78	0.60, 1.02	0.072	0.83	0.58, 1.19	0.300
SD and ST	0.72	0.48, 1.08	0.110	0.55	0.28, 1.03	0.073
All	0.71	0.53, 0.93	0.014	0.69	0.47, 1.02	0.059
Number of guidelines met						
None	Reference			Reference		
One	0.85	0.66, 1.09	0.200	0.77	0.55, 1.08	0.120
Two	0.76	0.59, 0.97	0.030	0.71	0.51, 1.00	0.047
Three	0.71	0.53, 0.93	0.014	0.69	0.47, 1.02	0.061

^aAdjusted for age, gender, parental myopia, parental education and family income.^

PA = physical activity; ST = screen time; SD = sleep duration; aOR = adjusted odds ratio; CI = confidence interval.

We examined the combined effects of meeting multiple guidelines on the risk of myopia and anisometropia (Table 5). Participants who met both the screen time and physical activity guidelines had a significantly reduced risk of myopia (aOR = 0.73, 95% CI = 0.56–0.97, *p* = 0.029) and demonstrated a significantly reduced odds of anisometropia (aOR = 0.60, 95% CI: 0.41–0.89, *p* = 0.011). Similarly, participants who met all three guidelines (PA, ST, and SD) were significantly less likely to develop myopia (aOR = 0.71, 95% CI: 0.53–0.93, *p* = 0.014) and showed a trend toward reduced risk of anisometropia (aOR = 0.69, 95% CI: 0.47–1.02, *p* = 0.059), compared to those who did not meet any of the guidelines. Participants who met two guidelines also had a significantly reduced risk of myopia (aOR = 0.76, 95% CI: 0.59–0.97, *p* = 0.030) and anisometropia (aOR = 0.71, 95% CI: 0.51–1.00, *p* = 0.047). Meeting only one guideline did not significantly affect the risk of either myopia or anisometropia.

## Discussion

This study examined the associations between adherence to the Canadian 24-h movement guidelines—encompassing physical activity (PA), screen time (ST), and sleep duration (SD)—and vision impairment, specifically myopia and myopic anisometropia, in a sample of children and adolescents from Shenzhen, China. The results provide valuable insights into how modern lifestyle behaviors, particularly those related to physical activity, screen time, and sleep, may influence visual health in children. The key findings highlight the potential role of reducing screen time and promoting physical activity in mitigating the risk of myopia, while associations with myopic anisometropia were less consistent.

### Adherence to 24-hour movement guidelines and risk of myopia

Our findings indicate that meeting the screen time guideline was significantly associated with a reduced risk of myopia, even when adjusted for potential confounders. Specifically, children who limited their recreational screen time to the recommended levels (≤ 2 h per day) had 14% lower odds of developing myopia compared to those who exceeded this limit. This result aligns with previous research indicating that prolonged near-work activities, including screen use, are a major contributor to the increasing prevalence of myopia in children and adolescents (17, 18, 23, 24). Excessive screen time likely contributes to myopia through increased near-work demand, reduced outdoor time, and increased digital eye strain, all of which have been implicated in the pathogenesis of myopia.

The protective effect of adhering to the physical activity guideline on myopia risk was borderline significant, suggesting that engaging in at least 60 min of moderate-to-vigorous physical activity per day may also play a role in reducing myopia risk. This aligns with evidence from prior studies (20–22, 38–40), which have consistently demonstrated that more time spent outdoors, often linked to physical activity, is associated with a lower risk of myopia onset and progression. Outdoor light exposure has been proposed as a potential mechanism for this protective effect, as it may slow down axial elongation of the eye (39, 43). Given that 80.47% of participants in this study met the PA guideline, further interventions promoting outdoor activity may be an effective public health strategy to prevent myopia.

Interestingly, adherence to the sleep duration guideline alone was not significantly associated with myopia in this study. While previous research has suggested a potential link between insufficient sleep and myopia due to disruptions in the circadian rhythm (44), the lack of significant findings in this study may be attributed to several factors, such as the study design, sample size, or inconsistencies in the definition of sleep duration across different studies. These variations could have influenced the observed associations between sleep duration and myopia risk in this population. However, it is worth noting that sleep could interact with other lifestyle factors, and further research is needed to fully understand the relationship between sleep and myopia.

When considering combined adherence to the movement guidelines, meeting both the PA and ST guidelines together significantly reduced the odds of myopia by 27%. Furthermore, participants who met all three guidelines (PA, ST, and SD) had the lowest risk of myopia, with a 29% reduction in odds compared to those who met none of the guidelines. This suggests that a balanced lifestyle, incorporating adequate physical activity, limited screen time, and sufficient sleep, may have a cumulative protective effect on visual health. These findings emphasize the importance of promoting adherence to all components of the movement guidelines as part of a holistic approach to myopia prevention.

### Adherence to 24-hour movement guidelines and risk of anisometropia

For myopic anisometropia, adherence to the ST guideline was significantly associated with a reduced risk (aOR = 0.78, 95% CI: 0.64–0.95, *p* = 0.015). This finding suggests that limiting screen time may help mitigate the risk of myopic anisometropia, potentially by reducing prolonged near-work activities that contribute to refractive disparities between the eyes.

Adherence to the PA guideline did not show a significant association with myopic anisometropia (aOR = 0.88, 95% CI: 0.70–1.10, *p* = 0.300). While PA is well-documented for its protective effect against myopia, its role in myopic anisometropia appears minimal, likely due to the structural and developmental nature of this condition.

Similarly, no significant association was observed between adherence to the SD guideline and myopic anisometropia (aOR = 1.04, 95% CI: 0.86–1.25, *p* = 0.700). This result aligns with findings for myopia, indicating that sleep duration may not serve as a strong independent predictor of myopic anisometropia.

When examining the combined effects of meeting multiple guidelines, adherence to both the PA and ST guidelines was significantly associated with a lower risk of myopic anisometropia (aOR = 0.60, 95% CI: 0.41–0.89, *p* = 0.011). However, adherence to all three guidelines (PA, ST, and SD) showed only a trend toward reduced risk without reaching statistical significance (aOR = 0.69, 95% CI: 0.47–1.02, *p* = 0.059).

These findings suggest that while limiting screen time and engaging in physical activity may reduce the risk of myopic anisometropia, the etiology of this condition likely involves factors beyond those captured by the 24-hour movement guidelines. Further research is needed to investigate the potential roles of genetic predisposition, early developmental influences, and other environmental factors in the development of myopic anisometropia.

### Implications for public health and myopia prevention

The findings of this study have important implications for public health strategies aimed at preventing vision impairment, particularly myopia, in children and adolescents. The significant protective effects of limiting screen time and promoting physical activity support the need for interventions targeting these behaviors. In rapidly urbanizing cities like Shenzhen, where children are increasingly exposed to digital screens for both educational and recreational purposes, efforts to reduce screen time may be crucial in addressing the rising rates of childhood myopia. Public health campaigns, school-based programs, and parental guidance should emphasize the importance of managing screen time and encouraging outdoor physical activity as part of daily routines.

Moreover, promoting adherence to the full set of 24-hour movement guidelines—balancing physical activity, screen time, and sleep—may offer the greatest benefits for preventing myopia and promoting overall health. While sleep alone did not emerge as a strong predictor of myopia risk, its role in supporting a balanced lifestyle should not be overlooked, as adequate sleep is essential for cognitive development, emotional well-being, and physical health in children.

### Strengths and limitations

This study has several strengths, including the use of a large sample size from a rapidly urbanizing region in China and the comprehensive assessment of adherence to the 24-hour movement guidelines. Additionally, the study adjusts for several important confounders, allowing for more accurate estimates of the associations between lifestyle behaviors and vision impairment.

However, there are also limitations to consider. First, the diagnostic criteria for myopia and anisometropia in this study were based on non-cycloplegic refraction, which has been validated as having high sensitivity and specificity for myopia screening among students (45). However, this approach may have led to a slight overestimation of myopia prevalence. Second, the cross-sectional design of the study limits the ability to establish causality between adherence to the movement guidelines and vision outcomes. Longitudinal studies are needed to confirm the temporal relationships between lifestyle behaviors and the development of myopia and anisometropia. Third, the use of self-reported measures for screen time and sleep duration may introduce recall bias, potentially underestimating or overestimating actual adherence to the guidelines. Objective measures of these behaviors, such as wearable activity trackers or screen time monitoring software, would strengthen future research. Finally, while our analysis adjusted for several key confounders, such as age, gender, parental myopia, and family socioeconomic status, we acknowledge that other potential confounding factors, such as unmeasured environmental influences, may have impacted the observed associations.

## Conclusion

In conclusion, this study demonstrates that adherence to the Canadian 24-hour movement guidelines, particularly the screen time (ST) component, is significantly associated with a reduced risk of myopia and myopic anisometropia in children and adolescents. Meeting both the ST and physical activity (PA) guidelines provided additional protective benefits, with the strongest effect observed for myopia when all three guidelines were met. These findings highlight the importance of promoting balanced lifestyle behaviors, especially limiting screen time and encouraging physical activity, to combat the rising prevalence of myopia and myopic anisometropia. Future interventions should focus on these modifiable factors while further exploring the complex etiology of myopic anisometropia.

## Data Availability

The original contributions presented in the study are included in the article/Supplementary materials, further inquiries can be directed to the corresponding author.
