# Supplementary material for: Adherence to the Canadian 24-hour movement guidelines and vision impairment in children and adolescents: a cross-sectional study
Source: Front Med (Lausanne). 2025 Feb 12;12:1523640. doi: 10.3389/fmed.2025.1523640 (PMC11860970; doi:10.3389/fmed.2025.1523640)
Supplement: Supplementary file 1 [file Table_1.docx]

**Supplementary file**

**Table S1.** The prevalence of different subtypes of anisometropia among study participants

| **Subtype*** | **Frequencies(n)** | **Percentage (%)** |
| --- | --- | --- |
| **Emmetropic** | 60 | 8.37 |
| **Myopic** | 575 | 80.20 |
| **Hyperopic** | 74 | 10.32 |
| **Antimetropic** | 8 | 1.12 |
| **Meridional** | 295 | 6.08 |
| **Simple meridional** | 207 | 70.17 |
| **Total** | 924 | 19.04 |

*The definition of different subtypes of anisometropia(1):

Spherical anisometropia (SA): absolute interocular differences in spherical equivalent refraction (SER)≥1.00D.

Emmetropic ~: SA, plus both eyes were emmetropic (1.00D < SER < +1.00D).

Myopic ~ : SA, plus both eyes were myopic (SER≤1.00D) or if one eye was myopic and the other was emmetropic.

Hyperopic ~ : SA, plus both eyes being hyperopic (SER≥+1.00D) or one eye being hyperopic and the other emmetropic.

Antimetropic ~: SA, plus one eye was myopic and the other hyperopic.

Meridional ~ (MA): or astigmatic anisometropia, absolute interocular differences in cylindrical diopters≥1.00D.

Simple meridional ~ : MA, plus absolute interocular differences in spherical equivalent refraction (SER)＜1.00D.

Total ~: SA plus MA.

1. Nunes AF, Batista M and Monteiro P. Prevalence of anisometropia in children and adolescents [version 4; peer review: 2 approved] F1000Research 2022, 10:1101 https://doi.org/10.12688/f1000research.73657.4

**Table S2.** Descriptive statistics of adherence to 24-hour movement guidelines and different subtypes of anisometropia

| **Exposures** | **Emmetropic** | | **p-value** |  | **Myopic** | | **p-value** |  | **Hyperopic** | | **p-value** |  | **Antimetropic** | | **p-value** |  | **Meridional** | | **p-value** |  | **Simple meridional** | | **p-value** |  | **Total anisometropia** | | **p-value** |
| --- | --- | --- | --- | --- | --- | --- | --- | --- | --- | --- | --- | --- | --- | --- | --- | --- | --- | --- | --- | --- | --- | --- | --- | --- | --- | --- | --- |
|  | **Yes** | **No** |  |  | **Yes** | **No** |  |  | **Yes** | **No** |  |  | **Yes** | **No** |  |  | **Yes** | **No** |  |  | **Yes** | **No** |  |  | **Yes** | **No** |  |
| **PA** |  |  | >0.999 |  |  |  | 0.082 |  |  |  | 0.555 |  |  |  | 0.192 |  |  |  | 0.495 |  |  |  | 0.530 |  |  |  | 0.106 |
| Yes | 49 (82%) | 3,858 (80%) |  |  | 447 (78%) | 3,460 (81%) |  |  | 62 (84%) | 3,845 (80%) |  |  | 5 (63%) | 3,902 (81%) |  |  | 233 (79%) | 3,674 (81%) |  |  | 163 (79%) | 3,744 (81%) |  |  | 726 (79%) | 3,181 (81%) |  |
| **ST** |  |  | 0.600 |  |  |  | <0.001 |  |  |  | 0.722 |  |  |  | 0.482 |  |  |  | 0.273 |  |  |  | 0.097 |  |  |  | 0.029 |
| Yes | 27 (45%) | 1,993 (42%) |  |  | 199 (35%) | 1,821 (43%) |  |  | 29 (39%) | 1,991 (42%) |  |  | 2 (25%) | 2,018 (42%) |  |  | 132 (45%) | 1,888 (41%) |  |  | 98 (47%) | 1,922 (41%) |  |  | 355 (38%) | 1,665 (42%) |  |
| **SD** |  |  | 0.795 |  |  |  | 0.422 |  |  |  | 0.077 |  |  |  | >0.999 |  |  |  | 0.587 |  |  |  | 0.354 |  |  |  | 0.659 |
| Yes | 28 (47%) | 2,143 (45%) |  |  | 248 (43%) | 1,923 (45%) |  |  | 41 (55%) | 2,130 (45%) |  |  | 4 (50%) | 2,167 (45%) |  |  | 127 (43%) | 2,044 (45%) |  |  | 86 (42%) | 2,085 (45%) |  |  | 407 (44%) | 1,764 (45%) |  |
| **Guidelines met** |  |  | 0.894 |  |  |  |  |  |  |  |  |  |  |  | 0.414 |  |  |  |  |  |  |  |  |  |  |  |  |
| None | 5 (8.3%) | 347 (7.2%) |  |  | 57 (9.9%) | 295 (6.9%) |  |  | 5 (6.8%) | 347 (7.3%) |  |  | 1 (13%) | 351 (7.2%) |  |  | 21 (7.1%) | 331 (7.3%) |  |  | 15 (7.2%) | 337 (7.3%) |  |  | 83 (9.0%) | 269 (6.8%) |  |
| PA only | 14 (23%) | 1,283 (27%) |  |  | 165 (29%) | 1,132 (26%) |  |  | 17 (23%) | 1,280 (27%) |  |  | 2 (25%) | 1,295 (27%) |  |  | 73 (25%) | 1,224 (27%) |  |  | 51 (25%) | 1,246 (27%) |  |  | 249 (27%) | 1,048 (27%) |  |
| ST only | 1 (1.7%) | 242 (5.0%) |  |  | 28 (4.9%) | 215 (5.0%) |  |  | 1 (1.4%) | 242 (5.1%) |  |  | 1 (13%) | 242 (5.0%) |  |  | 15 (5.1%) | 228 (5.0%) |  |  | 12 (5.8%) | 231 (5.0%) |  |  | 43 (4.7%) | 200 (5.1%) |  |
| SD only | 2 (3.3%) | 176 (3.7%) |  |  | 27 (4.7%) | 151 (3.5%) |  |  | 3 (4.1%) | 175 (3.7%) |  |  | 1 (13%) | 177 (3.7%) |  |  | 15 (5.1%) | 163 (3.6%) |  |  | 10 (4.8%) | 168 (3.6%) |  |  | 43 (4.7%) | 135 (3.4%) |  |
| PA and ST | 12 (20%) | 779 (16%) |  |  | 77 (13%) | 714 (17%) |  |  | 10 (14%) | 781 (16%) |  |  | 0 (0%) | 791 (16%) |  |  | 59 (20%) | 732 (16%) |  |  | 43 (21%) | 748 (16%) |  |  | 142 (15%) | 649 (17%) |  |
| PA and SD | 12 (20%) | 995 (21%) |  |  | 127 (22%) | 880 (21%) |  |  | 20 (27%) | 987 (21%) |  |  | 2 (25%) | 1,005 (21%) |  |  | 54 (18%) | 953 (21%) |  |  | 33 (16%) | 974 (21%) |  |  | 194 (21%) | 813 (21%) |  |
| ST and SD | 3 (5.0%) | 171 (3.6%) |  |  | 16 (2.8%) | 158 (3.7%) |  |  | 3 (4.1%) | 171 (3.6%) |  |  | 0 (0%) | 174 (3.6%) |  |  | 11 (3.7%) | 163 (3.6%) |  |  | 7 (3.4%) | 167 (3.6%) |  |  | 29 (3.1%) | 145 (3.7%) |  |
| All | 11 (18%) | 801 (17%) |  |  | 78 (14%) | 734 (17%) |  |  | 15 (20%) | 797 (17%) |  |  | 1 (13%) | 811 (17%) |  |  | 47 (16%) | 765 (17%) |  |  | 36 (17%) | 776 (17%) |  |  | 141 (15%) | 671 (17%) |  |
| **Number of guidelines met** |  |  | 0.666 |  |  |  | 0.006 |  |  |  | 0.566 |  |  |  | 0.576 |  |  |  | 0.967 |  |  |  | 0.993 |  |  |  | 0.084 |
| None | 5 (8.3%) | 347 (7.2%) |  |  | 57 (9.9%) | 295 (6.9%) |  |  | 5 (6.8%) | 347 (7.3%) |  |  | 1 (13%) | 351 (7.2%) |  |  | 21 (7.1%) | 331 (7.3%) |  |  | 15 (7.2%) | 337 (7.3%) |  |  | 83 (9.0%) | 269 (6.8%) |  |
| One | 17 (28%) | 1,701 (35%) |  |  | 220 (38%) | 1,498 (35%) |  |  | 21 (28%) | 1,697 (36%) |  |  | 4 (50%) | 1,714 (35%) |  |  | 103 (35%) | 1,615 (35%) |  |  | 73 (35%) | 1,645 (35%) |  |  | 335 (36%) | 1,383 (35%) |  |
| Two | 27 (45%) | 1,945 (41%) |  |  | 220 (38%) | 1,752 (41%) |  |  | 33 (45%) | 1,939 (41%) |  |  | 2 (25%) | 1,970 (41%) |  |  | 124 (42%) | 1,848 (41%) |  |  | 83 (40%) | 1,889 (41%) |  |  | 365 (40%) | 1,607 (41%) |  |
| Three | 11 (18%) | 801 (17%) |  |  | 78 (14%) | 734 (17%) |  |  | 15 (20%) | 797 (17%) |  |  | 1 (13%) | 811 (17%) |  |  | 47 (16%) | 765 (17%) |  |  | 36 (17%) | 776 (17%) |  |  | 141 (15%) | 671 (17%) |  |

**Table S3**. Association between meeting 24-hour movement guidelines and different subtypes of anisometropia§

| **Subtype** | **Predictor** | **aOR** | **95%CI** | **p-value** |
| --- | --- | --- | --- | --- |
| **Emmetropic** | PA | 1.10 | 0.59, 2.24 | 0.8 |
|  | ST | 1.11 | 0.65, 1.86 | 0.7 |
|  | SD | 1.06 | 0.63, 1.77 | 0.8 |
| **Myopic** | PA | 0.89 | 0.72, 1.10 | 0.3 |
|  | ST | 0.81 | 0.67, 0.98 | 0.028* |
|  | SD | 1.03 | 0.86, 1.23 | 0.8 |
| **Hyperopic** | PA | 1.21 | 0.67, 2.38 | 0.6 |
|  | ST | 0.91 | 0.56, 1.46 | 0.7 |
|  | SD | 1.56 | 0.98, 2.51 | 0.063. |
| **Antimetropic** | PA | 0.40 | 0.10, 2.00 | 0.2 |
|  | ST | 0.64 | 0.09, 2.87 | 0.6 |
|  | SD | 1.63 | 0.38, 7.03 | 0.5 |
| **Meridional** | PA | 0.93 | 0.70, 1.25 | 0.6 |
|  | ST | 1.18 | 0.93, 1.50 | 0.2 |
|  | SD | 0.95 | 0.75, 1.21 | 0.7 |
| **Simple meridional** | PA | 0.92 | 0.66, 1.31 | 0.6 |
|  | ST | 1.29 | 0.97, 1.72 | 0.076 |
|  | SD | 0.89 | 0.66, 1.18 | 0.4 |
| **Total anisometropia** | PA | 0.91 | 0.76, 1.09 | 0.3 |
|  | ST | 0.93 | 0.80, 1.08 | 0.3 |
|  | SD | 1.04 | 0.90, 1.20 | 0.6 |

§ adjusted for age, gender, parental myopia, parental education and family income.

Significance codes: “*”:< 0.05; “.”:< 0.1

**Table S4.** Combined effects of meeting 24-hour movement guidelines on different subtypes of anisometropia§

| **Subtype** | **Predictor** | **aOR** | **95%CI** | **p-value** |
| --- | --- | --- | --- | --- |
| **Emmetropic** |  |  |  |  |
|  | **Guidelines met** |  |  |  |
|  | None | Reference |  |  |
|  | PA only | 0.74 | 0.28, 2.32 | 0.6 |
|  | ST only | 0.26 | 0.01, 1.65 | 0.2 |
|  | SD only | 0.73 | 0.10, 3.46 | 0.7 |
|  | ST and PA | 1.02 | 0.37, 3.27 | >0.9 |
|  | SD and PA | 0.81 | 0.29, 2.59 | 0.7 |
|  | SD and ST | 1.11 | 0.22, 4.64 | 0.9 |
|  | All | 0.89 | 0.32, 2.91 | 0.8 |
|  | **Number of guidelines met** |  |  |  |
|  | None | Reference |  |  |
|  | One | 0.67 | 0.26, 2.06 | 0.4 |
|  | Two | 0.92 | 0.38, 2.77 | 0.9 |
|  | Three | 0.89 | 0.32, 2.91 | 0.8 |
| **Myopic** |  |  |  |  |
|  | **Guidelines met** |  |  |  |
|  | None | Reference |  |  |
|  | PA only | 0.80 | 0.58, 1.12 | 0.2 |
|  | ST only | 0.76 | 0.46, 1.22 | 0.3 |
|  | SD only | 1.01 | 0.60, 1.67 | >0.9 |
|  | ST and PA | 0.68 | 0.46, 0.99 | 0.040* |
|  | SD and PA | 0.86 | 0.61, 1.23 | 0.4 |
|  | SD and ST | 0.64 | 0.34, 1.14 | 0.150 |
|  | All | 0.72 | 0.49, 1.05 | 0.084. |
|  | **Number of guidelines met** |  |  |  |
|  | None | Reference |  |  |
|  | One | 0.82 | 0.60, 1.13 | 0.2 |
|  | Two | 0.77 | 0.56, 1.07 | 0.110 |
|  | Three | 0.72 | 0.49, 1.05 | 0.085. |
| **Hyperopic** |  |  |  |  |
|  | **Guidelines met** |  |  |  |
|  | None | Reference |  |  |
|  | PA only | 0.95 | 0.37, 2.92 | >0.9 |
|  | ST only | 0.32 | 0.02, 2.00 | 0.3 |
|  | SD only | 1.44 | 0.29, 6.01 | 0.6 |
|  | ST and PA | 0.97 | 0.34, 3.16 | >0.9 |
|  | SD and PA | 1.49 | 0.59, 4.54 | 0.4 |
|  | SD and ST | 1.35 | 0.27, 5.64 | 0.7 |
|  | All | 1.33 | 0.50, 4.18 | 0.6 |
|  | **Number of guidelines met** |  |  |  |
|  | None | Reference |  |  |
|  | One | 0.91 | 0.37, 2.75 | 0.8 |
|  | Two | 1.27 | 0.53, 3.77 | 0.6 |
|  | Three | 1.33 | 0.50, 4.20 | 0.6 |
| **Antimetropic** |  |  |  |  |
|  | **Guidelines met** |  |  |  |
|  | None | Reference |  |  |
|  | PA only | 0.57 | 0.05, 12.5 | 0.7 |
|  | ST only | 2.06 | 0.08, 53.1 | 0.6 |
|  | SD only | 2.99 | 0.11, 78.9 | 0.4 |
|  | ST and PA | 0.00 | / | >0.9 |
|  | SD and PA | 0.89 | 0.08, 19.6 | >0.9 |
|  | SD and ST | 0.00 | / | >0.9 |
|  | All | 0.79 | 0.03, 20.8 | 0.9 |
|  | **Number of guidelines met** |  |  |  |
|  | None | Reference |  |  |
|  | One | 0.93 | 0.13, 18.4 | >0.9 |
|  | Two | 0.48 | 0.04, 10.6 | 0.6 |
|  | Three | 0.79 | 0.03, 20.8 | 0.9 |
| **Meridional** |  |  |  |  |
|  | **Guidelines met** |  |  |  |
|  | None | Reference |  |  |
|  | PA only | 0.96 | 0.59, 1.63 | 0.9 |
|  | ST only | 1.07 | 0.53, 2.12 | 0.8 |
|  | SD only | 1.51 | 0.74, 2.99 | 0.2 |
|  | ST and PA | 1.35 | 0.81, 2.31 | 0.3 |
|  | SD and PA | 0.94 | 0.57, 1.62 | 0.8 |
|  | SD and ST | 1.12 | 0.51, 2.34 | 0.8 |
|  | All | 1.04 | 0.62, 1.82 | 0.9 |
|  | **Number of guidelines met** |  |  |  |
|  | None | Reference |  |  |
|  | One | 1.03 | 0.65, 1.72 | 0.9 |
|  | Two | 1.12 | 0.70, 1.85 | 0.7 |
|  | Three | 1.04 | 0.61, 1.82 | 0.9 |
| **Simple meridional** |  |  |  |  |
|  | **Guidelines met** |  |  |  |
|  | None | Reference |  |  |
|  | PA only | 0.95 | 0.54, 1.77 | 0.9 |
|  | ST only | 1.21 | 0.54, 2.64 | 0.6 |
|  | SD only | 1.41 | 0.60, 3.19 | 0.4 |
|  | ST and PA | 1.37 | 0.76, 2.59 | 0.3 |
|  | SD and PA | 0.81 | 0.44, 1.55 | 0.5 |
|  | SD and ST | 0.96 | 0.36, 2.33 | >0.9 |
|  | All | 1.10 | 0.60, 2.12 | 0.8 |
|  | **Number of guidelines met** |  |  |  |
|  | None | Reference |  |  |
|  | One | 1.03 | 0.60, 1.89 | >0.9 |
|  | Two | 1.04 | 0.61, 1.90 | 0.9 |
|  | Three | 1.10 | 0.60, 2.11 | 0.8 |
| **Total anisometropia** |  |  |  |  |
|  | **Guidelines met** |  |  |  |
|  | None | Reference |  |  |
|  | PA only | 0.81 | 0.61, 1.09 | 0.2 |
|  | ST only | 0.77 | 0.50, 1.16 | 0.2 |
|  | SD only | 1.14 | 0.74, 1.74 | 0.6 |
|  | ST and PA | 0.83 | 0.61, 1.13 | 0.2 |
|  | SD and PA | 0.87 | 0.65, 1.18 | 0.4 |
|  | SD and ST | 0.76 | 0.47, 1.21 | 0.3 |
|  | All | 0.83 | 0.61, 1.14 | 0.2 |
|  | **Number of guidelines met** |  |  |  |
|  | None | Reference |  |  |
|  | One | 0.84 | 0.64, 1.11 | 0.2 |
|  | Two | 0.85 | 0.64, 1.12 | 0.2 |
|  | Three | 0.83 | 0.61, 1.14 | 0.2 |

§ adjusted for age, gender, parental myopia, parental education and family income.

Significance codes: “*”:< 0.05; “.”:< 0.1
